# Swapping data: A pragmatic approach for enabling academic-industrial partnerships

**DOI:** 10.1177/20552076231172120

**Published:** 2023-05-08

**Authors:** Julia Kasprzak, Simon Frey, Hermann Oetlinger, C. Benedikt Westphalen, Nicole Erickson, Volker Heinemann, Daniel Nasseh

**Affiliations:** 1 Comprehensive Cancer Center Munich, 27192 University Hospital, LMU Munich, Munich, Germany; 2 123188 Roche Pharma AG, Grenzach-Wyhlen, Germany; 3 German Cancer Consortium (DKTK, partner site Munich), German Cancer Research Center (DKFZ), Heidelberg, Germany

**Keywords:** Health data, anonymization, pseudonymization

## Abstract

**Objectives:**

Academic institutions have access to comprehensive sets of real-world data. However, their potential for secondary use—for example, in medical outcomes research or health care quality management—is often limited due to data privacy concerns. External partners could help achieve this potential, yet documented frameworks for such cooperation are lacking. Therefore, this work presents a pragmatic approach for enabling academic-industrial data partnerships in a health care environment.

**Methods:**

We employ a value-swapping strategy to facilitate data sharing. Using tumor documentation and molecular pathology data, we define a data-altering process as well as rules for an organizational pipeline that includes the technical anonymization process.

**Results:**

The resulting dataset was fully anonymized while still retaining the critical properties of the original data to allow for external development and the training of analytical algorithms.

**Conclusion:**

Value swapping is a pragmatic, yet powerful method to balance data privacy and requirements for algorithm development; therefore, it is well suited to enable academic-industrial data partnerships.

## Introduction

Data have become one of the most valuable resources for research and development and can drive innovation and progress.^1^ Health data are no exception.^2,3^ Large networks, like the Patient-Centered Clinical Research Network^4^ in the US or the Medical-Informatics-Initiative in Germany,^5^ understand that potential and try to compile routinely collected data within “data lakes” or “data warehouses”.^6^ Similar attempts regarding large data collections of disease-specific data registries from rare diseases^7^ to prosthetics^8^—or, in our use case, oncology^9^—have been documented. In 2021, the Comprehensive Cancer Center Munich of the LMU-Hospital (CCCM-LMU) established a tumor documentation system containing data from more than 40,000 patients with close to 3000 data fields per case.^10^ Although internal usage for tasks such as certification, controlling, or even research is usually easy to accomplish and, in many cases, backed by federal laws (e.g., Bavarian Law of Hospitals—Article 27 (4–6)),^11^ providing even partial access to external researchers or industry partners is more difficult to achieve.

The three biggest challenges to data sharing among external researchers are data privacy protection, governance issues, and confidentiality. The first problem can be handled through the use of patient consent. Based on patient consent, data can be used prospectively for specific purposes. However, for data collected in routine care, such consent is often missing. Some hospitals and initiatives currently try to address this problem by implementing broad research consent to facilitate the use of personal data collected around inpatient care. Unfortunately, data collected before the implementation of an explicit patient consent process may be irrelevant for future research use.^12^ At least in Europe, based on the GDPR,^13^ the only solution to work with routine data in the absence of consent is anonymization.^14^

Accordingly, in order to use existing data, such data have to be altered to such a degree that re-identification of patients is factually impossible. As opposed to pseudonymization—both concepts are often confused—anonymization does not just rely on deleting the identifying information (IDAT: e.g., name, address). Depending on the context, this may not suffice, as identities can be reassembled quite easily by using non-identifying data fields within the medical data contents (MDAT).^15–19^ Anonymization focuses on the MDAT as well; thus, for complex datasets with many data fields, full anonymization (irreversible) is hard to achieve.^18,19^ However, restricting large data sets to a smaller subset of data by including only chosen data fields can fix this issue if methods such as k-anonymity, l-diversity, or t-closeness are properly applied.^15–17^ Nonetheless, because this approach is technically very challenging, delivering formal proof of factual anonymity remains hard, and due to alterations in data based on the given methods, important medical information might be lost.^18,19^

One of the biggest problems with this process is that any kind of data manipulation can potentially introduce a given bias, yet unknown items within the full data set might be important predictors—for example, when calculating a prognostic model. Artificial intelligence (AI) or machine learning has the capacity to identify such items, but if the data are thinned out prior to reaching the algorithm, generation of such prognostic models might not succeed.

In addition, data-driven research relies heavily on access to complete datasets to make full use of its potential. The so-called IT assessments often result in the creation of data dictionaries to document the semantics and ontology of data fields and objects. Unfortunately, however, this rarely provides enough information to gain a good overview of the data. Therefore, information about the completeness of data, as well as data types and contents, might be just as important. For example, if a team wants to analyze the performance status (ECOG) of a patient following a specific therapy, they might be misled by a data dictionary that merely states such a data field is generally included within the given data set. As is often the case with routine data, the problem might be that the ECOG^20^ is only available for a small subpopulation; hence, even with a well-documented data dictionary, adequate planning for statistical analyses may be impeded due to incomplete data.

All of these challenges lead to a situation in which valuable data often remains unused. Cooperation between academic and industrial partners can be especially problematic, as data privacy concerns can be very harmful to the reputation of both partners.^21^ Thus, out of fear and missing alternatives, many collaborative opportunities are missed. Particularly in the health care sector, the collaboration between academic and industry research partners opens the door for product innovation and technological progress. Research institutions are usually on the frontlines of data-related topics such as AI or deep learning; however, their achievements often lack an applied perspective. Conversely, industry partners tend to need guidance on what types of data to sample and which outcomes to analyze. For example, to enable molecularly guided drug therapy in oncology patients (which is challenging due to the complexity of cancer's genetic variants), a pharmaceutical manufacturer might want to understand the relationship between specific biomarkers and medical outcome indicators. Thus, research is needed to model such causal relations and to predict an optimal therapeutic strategy. This represents a valuable collaborative project for both academic and industry partners, and one from which patients, in particular, can benefit from the results and progress. Finally, while there are already companies^22,23^ working with hospitals to improve clinical trial recruitment by utilizing hospital data, this situation remains rare and might shift control of the data and governance issues away from the academic partner's site and to the industrial partner(s).

In this study, we aimed to find an appropriate solution to the problems outlined above. To this end, the CCC-LMU (as acting research institute) experimentally designed a pragmatic method to allow information and data sharing while upholding a maximum level of privacy protection without giving up control of any data. The shared data cannot be used to create any meaningful analytics; instead, it serves as a synthetic data set with an identical structure. Hence, algorithms developed on this data set will yield true results when applied to the true and structurally similar real dataset. The methods and details of the organizational pipeline are presented in the following sections.

## Methods

The main objective of the technical implementation is the creation of a collaborative data set that is regarded as completely anonymous. Henceforth, this data set will be referred to as “swapping data set”. This data set enables external partners to prepare analytical models within their own IT/research environment. Those models can then be applied to the real (nonanonymized) data set to generate meaningful results. Within this pipeline, the real data record is never shared between partners.

### Extracting data field information from the source data

**Figure 1.**
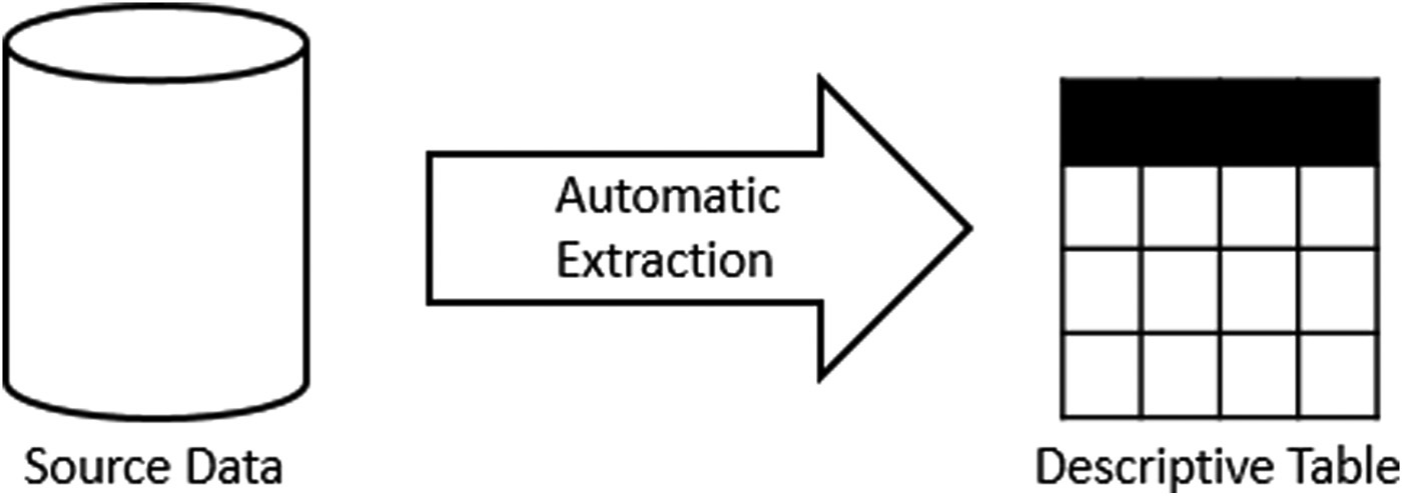
A descriptive table is generated by automatically extracting all table and parameter names from the source data sets and writing those into one comprehensive table.

Initially, to create the anonymized data set, a descriptive table is automatically generated with all column names (fields) extracted from all data tables of the internal partner's relational data sources (Figure 1). In the local scenario, the source data are CCCM-LMU's local tumor documentation data set and the local molecular pathological database (MolPath) governed by the molecular tumor board. The descriptive table that is generated serves as a data record description, with the first column containing the extracted system column names and the second column containing the system table names (Table 1).

**Table 1.** Data description with descriptions (column 3) and planned actions for each field (column 4).

Table name	Field name	Description	Measure
TZES	TZ_LOEKZ	Deletion indicator	No Action
TZVS	TZ_P_PID	Patient ID	Pseudonymization
TZTH	TZTH_A_BDD	Start of therapy	Date randomization
NPAT	Nname	Last name	Deletion
3CTDOKU	MANDT	Table 3CTDOKU will not be considered.	
…	…	…	…

### Adding description and evaluation in terms of privacy protection

All fields and source tables were described manually in the data record description (third column, Table 1) to provide a comprehensive understanding of the source data. In addition to manually adding a description for each field, a manual evaluation of privacy protection (meaning how the given field should be handled in the upcoming data transformation) was added (fourth column, Table 1). The following classifications for this evaluation were created: No Action, Deletion, Pseudonymization, and Date Randomization. No Action means that a given field will not be altered during the preparatory steps of creating the anonymized data set. By using the deletion flag, sensitive or unnecessary data, such as the patient's name and address, were marked to be completely deleted from the data record. Fields that have to remain in the data set but that contain sensitive markers, such as identifiers that connect the data tables, were flagged to be pseudonymized. Affected identifiers in the local use case were patient ID, tumor ID, and document ID. All date fields, which can be possibly Quasi-Identifiers,^24^ were flagged to be altered within a range between ± 3 and 6 days.

Additionally, some tables that were originally included in the tumor documentation software were completely excluded from the anonymized data set. These were system tables or tables that should not be passed on. In the data record description, these tables were specifically marked (third column, Table 1).

### Creation of a swapping data set

The data description as detailed in the above sections, specifically the evaluation flags (fourth column, Table 1), was used to automatically control and direct the technical anonymization processes (see Sampling and Swapping section). The program that was used to create the anonymized data set in the local use case is based on Java 1.8 and was only executed under the supervision of the internal partners from the research institute. In the given scenario, the users were exclusively employees of the CCCM-LMU. Access to and work with the real data set is reserved exclusively for internal hospital employees.

#### Implementation of anonymization measures

As previously described, if the deletion flag within the descriptive table is set, a deletion method completely erases all values from the respective column (Table 2). In the case of a pseudonymization flag, a respective method pseudonymizes all of the column's values using the SHA-3 hashing algorithm (Table 3).^24,25^ In order to secure the hash codes against dictionary attacks,^26^ the values are concatenated with a secret salt.^27^ The salt is generated as a random 16-byte value that is newly generated each time the program is used but is uniform across the data set. Since the salt is generated randomly when it is created, and since no key lists are stored, we can assume real, nonreversible, one-way encryption.

**Table 2.** Exemplary changes between the original data set and the swapping data set when the “Deletion mark” (column 4, Table 1) within the descriptive table is set for a given field. The marked field values will be deleted.

Original data set		Swapping data set	
First name	Last name	First name	Last name
Max	Mustermann	-	-
Daniel	Nasseh	-	-
Julia	Kasprzak	-	-
…	…	…	…

**Table 3.** Exemplary changes between the original data set and the swapping data set when the “Pseudonymization mark” (column 4, Table 1) within the descriptive table is set for a given field. A pseudonymization via SHA-3 and secret salt will be applied to the field.

Original data set		Swapping data set	
Patient ID	Tumor ID	Patient ID	Tumor ID
0022113344	00123	Ab345dTpk09c	96frmsDu89g
0012345678	00444	Hde967JmePdx	frG65s0heWB
0025836910	00771	whg43mssfOR7	1FwmyRF589
…	…	…	…

In our local use case, within the date randomization method, date values in the source data differ in two formats. The first format is “DD.MM.YYYY,” while in the second format, the date values are written in three separate columns: [DD][MM][YYYY].

In the first case example, dates are shifted by adding a random number between (±3) and (±6) days. The random number is calculated anew for each value from the respective column (Table 4). Both the salt for the pseudonymization and the random number for the date randomization were generated by using Java library's java.security package, which can also generate cryptographically secure randomization.^28^

**Table 4.** Exemplary changes between the original data set and the swapping data set when the “Randomization mark” (column 4, Table 1) within the descriptive table is set for a given field. In the case of combined dates, the affected field values will be randomized from (±3) to (±6) days.

Original data set	Swapping data set
Diagnosis date	Diagnosis date
01.04.2021	05.04.2021
16.07.2014	11.07.2014
27.12.2015	01.01.2016
…	…

In the second case example, in order to use date randomization, the three columns were concatenated so that the value now has the typical date format “DD.MM.YYYY.” Next, a random number was added, this time from the range of (±2) to (±10) days for each date value. The values were then shifted by the number of days and distributed to the three adjacent columns (Table 5).

**Table 5.** Exemplary changes between the original data set and the swapping data set when the “Randomization mark” (column 4, Table 1) within the descriptive table is set for a given field. In case of combined dates, the affected field values will be randomized from (±2) to (±)10 days.

Original data set			Swapping data set		
Diagnosis date Day	Diagnosis date Month	Diagnosis date Year	Diagnosis date Day	Diagnosis date Month	Diagnosis date Year
25	04	2018	30	04	2018
10	01	2014	06	01	2014
19	08	2020	10	08	2020
…	…	…	…	…	…

#### Sampling and Swapping

After transforming the data according to the preparatory anonymization steps, the value-swapping method was applied. This is the most important and, simultaneously, the most destructive step. Value swapping is an approach well-suited for anonymizing high-dimensional data. It essentially creates fake tuples by randomly permuting elements in each column of a data table. This breaks the linking between columns, which prevent any reestablishment of a link between the original information and natural persons. In doing so, almost all data connections are disrupted.

Cryptographically, true randomization should be used for this step. In our local use case, the java.security package was used. To preserve some of the information specific to disease classes, values were permuted within entity groups. Therefore, an entity group was composed of all observations with the same 3-digit ICD-10 code. Groups of less than 25 observations were merged with other small groups to ensure a high level of anonymity (Table 6).

**Table 6.** Data will be swapped within each individual column and according to an ICD-10 code. If there are fewer than 25 entries for an ICD-10 code, then these entries will be swapped in a group of all entries belonging to an ICD-10 code with fewer than 25 entries.

Original data set				Swapping data set			
Patient ID	Diagnosis date	ICD-10 Code	Tumor ID	Patient ID	Diagnosis date	ICD-10 Code	Tumor ID
0022113344	01.04.2021	C50.4	00123	Hde967JmePdx	01.01.2016	C50.9	frG65s0heWB
0012345678	16.07.2014	C50.8	00444	Ab345dTpk09c	05.04.2021	C50.4	1FwmyRF589
0025836910	27.12.2015	C50.4	00771	whg43mssfOR7	15.07.2012	C50.4	96frmsDu89g
0012233445	10.07.2012	C50.9	00258	medKI52eofG9	11.07.2014	C50.8	rdzre6ge2wGh
0015897463	15.06.2013	C34.9	00345	dj4ZbPebt6h8	02.04.2008	C34.9	tg5whgGht5jk
0025478931	08.02.2019	C34.1	00159	a6rkjvTfk2gT	11.06.2013	C34.9	tg5o8541sgjR
0014513145	26.04.2020	C34.2	00648	fw3972gaZDtc	30.04.2020	C34.1	Jktrhbr5e4GT
0011473695	31.03.2008	C34.9	00726	wSnge5b58swu	06.02.2019	C34.2	htr5r1jawFJn
…	…	…	…	…	…	…	…

Subsequently, and as an additional security measure, 30% of the rows in each table were randomly deleted (Table 7). The swapping data set with transformed values is saved in exactly the same format as the input tables.

**Table 7.** Sampling randomly deletes data entries and changes the frequencies between the original data set and the swapping data set.

Original data set				Swapping data set			
Patient ID	Diagnosis date	ICD-10 Code	Tumor ID	Patient ID	Diagnosis date	ICD-10 Code	Tumor ID
0022113344	01.04.2021	C50.4	00123	Hde967JmePdx	01.01.2016	C50.9	frG65s0heWB
0012345678	16.07.2014	C50.8	00444	a6rkjvTfk2gT	11.06.2013	C34.9	tg5o8541sgjR
0025836910	27.12.2015	C50.4	00771	medKI52eofG9	11.07.2014	C50.8	rdzre6ge2wGh
0012233445	10.07.2012	C50.9	00258	wSnge5b58swu	06.02.2019	C34.2	htr5r1jawFJn
0015897463	15.06.2013	C34.9	00345	whg43mssfOR7	15.07.2012	C50.4	96frmsDu89g
0025478931	08.02.2019	C34.1	00159	fw3972gaZDtc	30.04.2020	C34.1	Jktrhbr5e4GT
0014513145	26.04.2020	C34.2	00648				
0011473695	31.03.2008	C34.9	00726				
…	…	…	…	…	…	…	…

### Organizational pipeline

**Figure 2.**
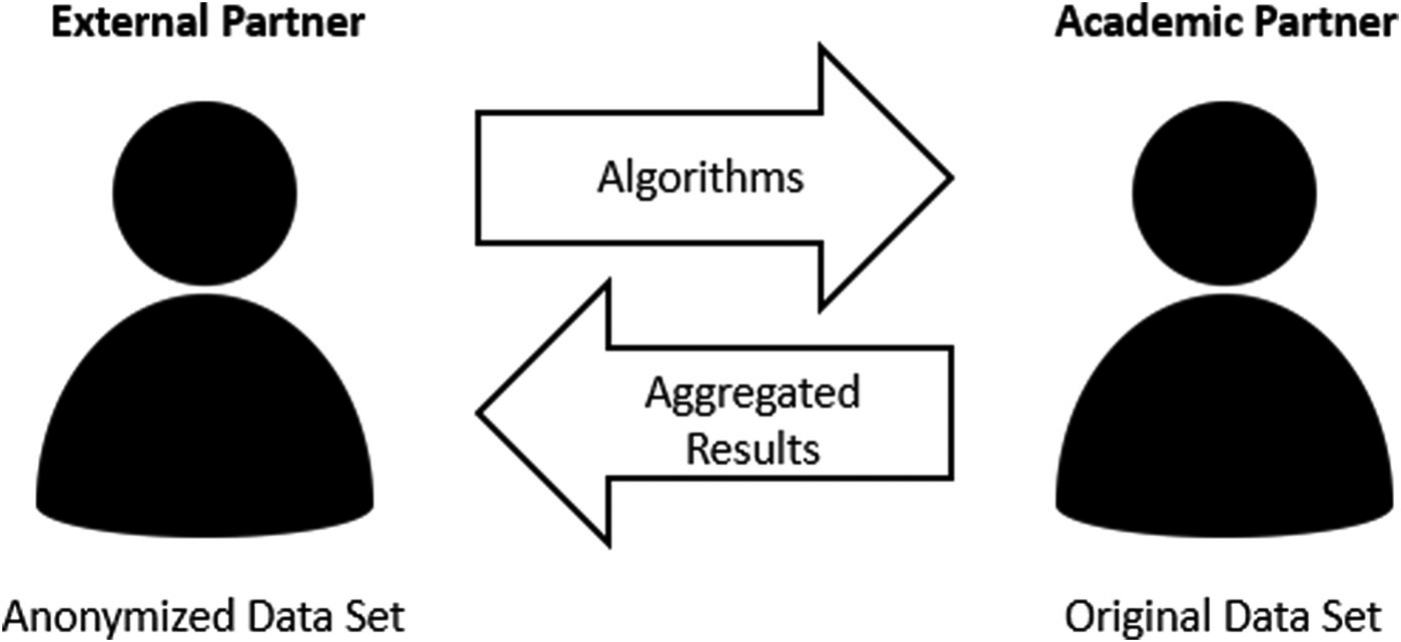
Organizational flow: Partners are given the swapping data set, based on which they can develop algorithms that will run but produce wrong results. Then, the algorithms can be directly applied to the original data under the control of the internal center. Thus, the control always remains at the site of the academic partner. After approving the aggregated results, the academic partner can freely access them and give them to the external partner.

The resulting collaborative data set can potentially be shared with external partners while upholding data protection laws and regulations. Partners are able to implement programs and analysis methods on the swapping data set. The externally created programs can then be implemented on-site and be used to generate real results. Results can then be shared with the industrial partner. The organizational control in this circle always remains at the hospital (Figure 2).

## Results

**Figure 3.**
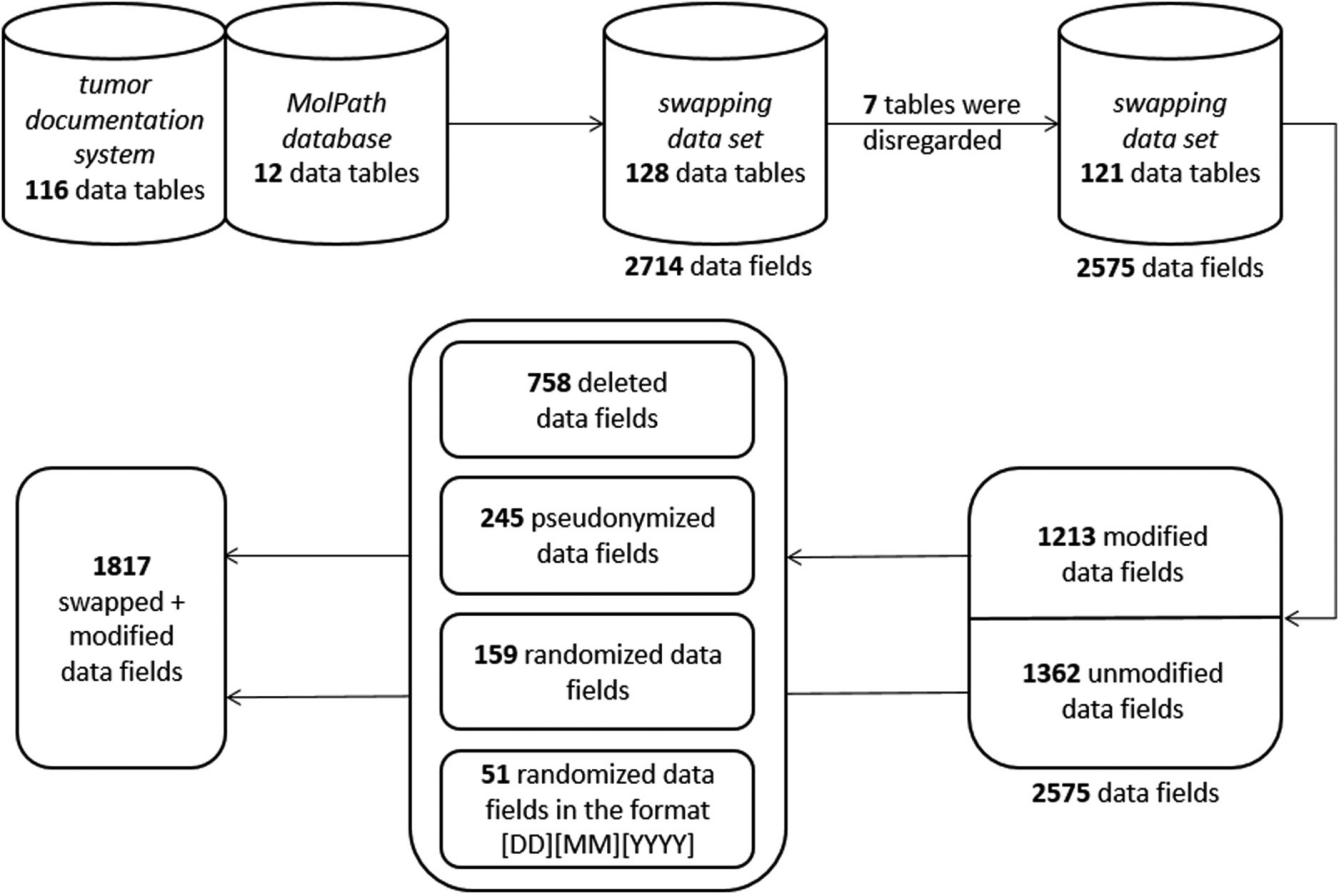
The illustration shows the stepwise application of the model on the local data sets displaying the count and changes in regard to data tables, data fields and modifications.

Although the main result of this work is the model of the pragmatic approach itself (according to methods), we use the section of results to display the count of modifications in regard to data tables and data fields after applying the model on the two given local data sets. Figure 3 summarizes these counts in accordance with the current step.

A total of 128 tables containing 2714 data fields were manually described and evaluated in terms of privacy protection in the data record description. As this step is of significant importance, it was performed by two employees in a time span of multiple weeks. Twelve of the 128 tables originated from the MolPath database, while the remaining 116 tables displayed the contents of CCCM-LMU's tumor documentation system. Seven tables were disregarded, as they contained technical information and were of no interest to the project.

The program for creating the swapping data set was implemented with Java 1.8. Both the source code of the program and the data record description were sent to LMU's data privacy protection commissionaire, where they were evaluated and cleared. Thus, the new swapping data set consisting of 121 tables was created.

Out of 2714 data fields, a total of 1213 fields were modified according to the predefined anonymization methods and the data record description. Fields with unknown meaning were marked to be truncated. The swapping data set was shared with an external partner in a secure, local environment. To validate its utility, the swapping data was used to create algorithms that could identify specific trial cohorts, which, when applied to the original data set, yielded the expected result. However, the swapping data set alone, independent of the original data, cannot be used to create meaningful results.

## Discussion

The given swapping data set should be considered to be fully anonymized. Identifying data fields that directly enable patient identification, such as family name, have been completely removed. This is referred to as formal anonymization.^14^

Other fields that could identify a patient could be data fields with a specific date (quasi-identifiers).^29^ In this case, all fields containing a date have been randomized. Given fields were flagged and double-checked over a span of multiple weeks, and fields of unknown meaning were marked for deletion.

One remaining danger of re-identifying a patient is the way in which different data fields belonging to one patient might relate to one another. For example, the combination of two rare diseases belonging to one case might hint at, or even reveal, the original identity of a patient. This problem can usually be solved on smaller data sets by applying generalization and grouping in regard to k-anonymity, but it is harder to achieve in larger data sets because of the combinatory opportunities.

Another example that cannot be solved through k-anonymity is the occurrence of unusual treatment patterns. A patient who was operated on an unusual number of times reveals a unique and distinct treatment pattern and, therefore, could be recognized by someone who kept track of the patient's hospital visits (background information) even if the dates (quasi-identifiers) of the visits were altered. This problem is often overlooked when checking for factual anonymity, but it is solved in our use case.

In our method, all general relations and patterns are broken completely apart. Currently, there does not seem to be any attack scenario resulting in the re-identification of a patient, as the relations in our original micro-data are completely disassembled and reassembled in a randomized order. One remaining security flaw might be the impossibility of true randomness, hence, the danger of reversing the randomization process.^30^ To avoid this danger, cryptographically secure algorithms, upheld as irreversible by current technological standards, were used (java.security).^28^

Although the whole approach results in high data privacy protection, the data itself cannot be used to create any meaningful analytics. However, the data set still holds some information that can be of significant value for a cooperating partner. The partner gains information about the exact structure of the data set and the contents of the data fields, as well as an understanding of the frequencies and distribution of values within each individual data field. This is especially important when working with incomplete data. For example, if one wants to include the performance status in an analysis, it is important to know whether it has been documented sufficiently. Thus, the swapping data set helps researchers check the completeness of the information in general. Furthermore, it helps researchers identify if the contents of a data field are sufficient for a desired analysis, as some parts (e.g., specific classes of disease) of the cohort might be better documented than others. This is exactly the case in terms of performance status, which might be useful for some analyses and too incomplete for others.

The fact that some parts of the data are documented better than other parts is, in general, a problem. For example, in terms of data quality at the CCCM-LMU, we usually have highly validated data coming from certified centers (distinct ICD-10 codes), while other kinds of cancers might be prone to missing data. To improve the swapping data set in that regard, we swapped prespecified tables within specific ICD-10 groups. Further disaggregation of groups would allow for even more insights, but it could also potentially compromise the quality of the anonymization. With only one group though, disease-related information can be conserved to a small degree, and the size of the groups can be kept sufficiently high (k = 25).

In addition, the frequencies of applied diagnostics and therapies would likely be of interest to industry partners. This can be a problem in terms of confidentiality and potentially sharing hospital secrets. To tackle this issue, we set up pseudonymization on fields we were concerned about. However, by applying expert knowledge and reviewing the frequencies, this pseudonymization could potentially, and with great effort, be reversed. Although we do not consider this issue to be too much of a problem, we still sampled all data (1/3 was randomly deleted); hence, we changed the original frequencies so that potential partners cannot know the exact frequencies at the sites.

As a limitation, the system has not yet been officially evaluated or certified by privacy protection experts (e.g., the Technology, Methods, and Infrastructure for Networked Medical Research [TMF e.V.] or the Federal Office for Information Security [BSI]),^31,32^ which is a step that could be considered in the future. Our current plans are to test the approach in the context of the Roche-Collaboration (ICON) project.^33^

The local ethics commission was contacted regarding this project. As this work presents a technical model for generating irreversible anonymous data based on retrospective data cohorts without any further intervention, the project was deemed to not require an ethics vote.

## Conclusion

We created a method that pragmatically allows academic and external partners to collaborate on data projects. The core of this system shuffles the original data, enables the swapped data to be analyzed, and creates results based on the original data while enabling the original data holders to retain full control of the original data. The solution offers a pragmatic way to share data with a strong focus on data privacy protection.

## References

[1] Brown FK Maliski E Waller C . Data is the currency of R&D, and that currency is now generated and traded globally. Curr Opin Drug Discov Dev 2010; 13: 275–278. 20464800

[2] Han A Isaacson A Muennig P . The promise of big data for precision population health management in the US. Public Health 2020; 185: 110–116. 32615477 10.1016/j.puhe.2020.04.040

[3] Rüping S . Big data in medizin und gesundheitswesen. Bundesgesundheitsblatt Gesundheitsforschung Gesundheitsschutz 2015; 58: 794–798. 26063521 10.1007/s00103-015-2181-y

[4] Pletcher MJ Forrest CB Carton TW . PCORNet's collaborative research groups. Patient Relat Outcome Meas 2018; 9: 91–95. 29467584 10.2147/PROM.S141630 PMC5811180

[5] Semler SC Wissing F Heyder R . German Medical informatics initiative. Methods Inf Med 2018; 57: e50–e56. 30016818 10.3414/ME18-03-0003 PMC6178199

[6] Pavlenko E Strech D Langhof H . Implementation of data access and use procedures in clinical data warehouses. A systematic review of literature and publicly available policies. BMC Med Inform Decis Mak 2020; 20: 157. 32652989 10.1186/s12911-020-01177-z PMC7353743

[7] Bilton D Caine N Cunningham S , et al. Use of a rare disease patient registry in long-term post-authorisation drug studies: a model for collaboration with industry. Lancet Respir Med 2018; 6: 495–496. 29735358 10.1016/S2213-2600(18)30192-9

[8] Kamrad I Söderberg B Örneholm H , et al. SwedeAmp-the Swedish amputation and prosthetics registry: 8-year data on 5762 patients with lower limb amputation show sex differences in amputation level and in patient-reported outcome. Acta Orthop 2020; 91: 464–470. 32316805 10.1080/17453674.2020.1756101 PMC8023884

[9] Thong MS Mols F Stein KD , et al. Population-based cancer registries for quality-of-life research: a work-in-progress resource for survivorship studies? Cancer 2013; 119: 2109–2123. 23695923 10.1002/cncr.28056

[10] Nasseh D Schneiderbauer S Lange M , et al. Optimizing the analytical value of oncology-related data based on an in-memory analysis layer: Development and assessment of the Munich online comprehensive cancer analysis platform. J Med Internet Res 2020; 22: e16533. 32077858 10.2196/16533 PMC7195671

[11] Bayerisches Krankenhausgesetz (BayKrG): Art. 27, https://www.gesetze-bayern.de/Content/Document/BayKrG-27 (2007, accessed 19 August 2021).

[12] Maloy JW Bass PF III . Understanding broad consent. Ochsner J 2020; 20: 81–86. 32284687 10.31486/toj.19.0088 PMC7122261

[13] General Data Protection Regulation (GDPR), https://gdpr-info.eu (2018, accessed 25 August 2021).

[14] Research Data Centres of the Statistical Offices of the Federation and the Federal States. Anonymity. https://www.forschungsdatenzentrum.de/en/anonymity (accessed 25 August 2021).

[15] Sweeney L . k-anonymity: a model for protecting privacy, international journal of uncertainty, fuzziness and knowledge-based systems. World Scientific 2002; 10: 557–570.

[16] Machanavajjhala A Kifer D Gehrke J , et al. l-diversity: Privacy beyond k-anonymity. *22nd International Conference on Data Engineering (ICDE'06)* 2006; 24.

[17] Li N Li T Venkatasubramanian S . t-Closeness: Privacy beyond k-anonymity and l-diversity. *23rd International Conference on Data Engineering* 2007; 106–115.

[18] Nasseh D . The mishandling of anonymity in terms of medical research within the general data protection regulation. Stud Health Technol Inform 2020; 272: 43–46. 32604596 10.3233/SHTI200489

[19] Salas J Domingo-Ferrer J . Some basics on privacy techniques, anonymization and their big data challenges. Math Comput Sci 2018; 12: 263–274.

[20] ECOG-Acrin cancer research group. ECOG Performance Status Scale, https://ecog-acrin.org/resources/ecog-performance-status (accessed 19 August 2021).

[21] Kusiak A . Smart manufacturing must embrace big data. Nature 2017; 544: 23–25. DOI: 10.1038/544023a. 28383012

[22] Tran Q Warren JL Barrett MJ , et al. An evaluation of the utility of big data to supplement cancer treatment information: Linkage between IQVIA pharmacy database and the surveillance, epidemiology, and end results program. J Natl Cancer Inst Monogr 2020; 2020: 72–81. 32412073 10.1093/jncimonographs/lgz036 PMC7868033

[23] Clinerion Real World Data Solutions, https://www.clinerion.com/index.html (accessed 19 August 2021).

[24] Rajendran K Jayabalan M Rana ME . A study on k-anonymity, l-diversity, and t-closeness techniques focusing medical data. Int J Comput Sci Netw Secur 2017; 17: 172–177.

[25] Sklavos N . Towards to SHA3 hashing standard for secure communications: On the hardware evaluation development. IEEE Latin Am Trans 2012; 10: 1433–1434.

[26] Raza M Iqbal M Sharif M , et al. A survey of password attacks and comparative analysis on methods for secure authentication. World Appl Sci J 2012; 19: 439–444.

[27] Rathod U Sonkar M Chandavarkar BR . An experimental evaluation on the dependency between one-way hash functions and salt. 2020 *11th International Conference on Computing, Communication and Networking Technologies (ICCCNT)* 2020; 1–7.

[28] Java API specification, Class SecureRandom, https://docs.oracle.com/javase/8/docs/api/java/security/SecureRandom.html (accessed 19 October 2021).

[29] Lee YJ Lee KH . What are the optimum quasi-identifiers to re-identify medical records? 2018 20th International Conference on Advanced Communication Technology 2018: 1025–1033.

[30] Calude CS . Quantum randomness: from practice to theory and back. Incomput Journeys Beyond Turing Barrier 2017: 169–181.

[31] TMF - Technology, Methods, and Infrastructure for Networked Medical Research, https://www.tmf-ev.de/ (accessed 19 August 2021).

[32] BSI - Bundesamt für Sicherheit in der Informationstechnik, https://www.bsi.bund.de/ (accessed 19 August 2021).

[33] Roche Deutschland Holding GmbH, Reelle Chancen für Patienten real machen: Das ICON-Projekt, https://www.roche.de/aktuelles/blog/das-icon-projekt/ (2020, accessed 19 August 2021).

